# Dexmedetomidine improved renal function in patients with severe sepsis: an exploratory analysis of a randomized controlled trial

**DOI:** 10.1186/s40560-019-0415-z

**Published:** 2020-01-02

**Authors:** Tsuyoshi Nakashima, Kyohei Miyamoto, Nozomu Shima, Seiya Kato, Yu Kawazoe, Yoshinori Ohta, Takeshi Morimoto, Hitoshi Yamamura, DESIRE Trial Investigators, Masaou Tanaka, Tomonori Yamamoto, Akihiro Fuke, Atsunori Hashimoto, Hiroyuki Koami, Satoru Beppu, Yoichi Katayama, Makoto Itoh

**Affiliations:** 1 Department of Emergency and Critical Care Medicine, Wakayama Medical University, 811-1, Kimiidera, Wakayama City, Wakayama Japan; 2 Division of Emergency and Critical Care Medicine, Tohoku University Graduate School of Medicine, 1-1, Seiryo-machi, Aoba-ku, Sendai City, Japan; 3 Dividion of General Medicine, Department of Internal Medicine, Hyogo College of Medicine, 1-1, Mukogawa-cho, Nishinomiya City, Japan; 4 Department of Clinical Epidemiology, Hyogo College of Medicine, 1-1, Mukogawa-cho, Nishinomiya City, Japan; 5 Osaka Prefecture Nakakawachi Critical Care and Emergency Center, 3-4-13, Nishiiwata, Higashiosaka City, Japan

**Keywords:** Dexmedetomidine, Sequential organ failure assessment score, Acute kidney injury, Septic shock, Sedation

## Abstract

**Background:**

Dexmedetomidine has been reported to improve organ dysfunction in critically ill patients. In a recent randomized controlled trial (Dexmedetomidine for Sepsis in Intensive Care Unit (ICU) Randomized Evolution [DESIRE]), we demonstrated that dexmedetomidine was associated with reduced mortality risk among patients with severe sepsis. We performed this exploratory sub-analysis to examine the mechanism underlying improved survival in patients sedated with dexmedetomidine.

**Methods:**

The DESIRE trial compared a sedation strategy with and without dexmedetomidine among 201 mechanically ventilated adult patients with sepsis across eight ICUs in Japan. In the present study, we included 104 patients with Acute Physiology and Chronic Health Evaluation II (APACHE II) scores of ≥ 23 (54 in the dexmedetomidine [DEX] group and 50 in the non-dexmedetomidine [non-DEX] group). Initially, we compared the changes in the sequential organ failure assessment (SOFA) scores from the baseline within 6 days after randomization between groups. Subsequently, we evaluated the variables comprising the organ component of the SOFA score that showed relevant improvement in the initial comparison.

**Results:**

The mean patient age was 71.0 ± 14.1 years. There was no difference in the median APACHE II score between the two groups (29 [interquartile range (IQR), 25–31] vs. 30 [IQR, 25–33]; *p* = 0.35). The median SOFA score at the baseline was lower in the DEX group (9 [IQR, 7–11] vs. 11 [IQR, 9–13]; *p* = 0.01). While the renal SOFA subscore at the baseline was similar for both groups, it significantly decreased in the DEX group on day 4 (*p* = 0.02). During the first 6 days, the urinary output was not significantly different (*p* = 0.09), but serum creatinine levels were significantly lower (*p* = 0.04) in the DEX group. The 28-day and in-hospital mortality rates were significantly lower in the DEX group (22% vs. 42%; *p* = 0.03, 28% vs. 52%; *p* = 0.01, respectively).

**Conclusion:**

A sedation strategy with dexmedetomidine is associated with improved renal function and decrease mortality rates among patients with severe sepsis.

**Trial registration:**

This trial was registered on ClinicalTrials.gov (NCT01760967) on January 1, 2013.

## Background

Dexmedetomidine is a sedative drug that has a unique mechanism of action. It is a selective α_2_-adrenergic agonist, unlike the more common gamma-aminobutyric acid receptor agonists such as midazolam and propofol [1]. Previous studies suggest that dexmedetomidine prevents delirium and enables clinicians to communicate with patients [1, 2]. Beyond its quality of improving sedation, dexmedetomidine may attenuate inflammatory reactions and protect against organ dysfunction such as acute kidney injury and liver dysfunction [3–6].

A previous randomized controlled trial reported that dexmedetomidine was associated with a reduced mortality rate among patients with sepsis [7]. Moreover, a recent randomized controlled trial that enrolled mechanically ventilated patients with sepsis, the Dexmedetomidine for Sepsis in Intensive Care Unit (ICU) Randomized Evolution (DESIRE) trial, showed that dexmedetomidine improves survival in the subgroup with more severe sepsis with an Acute Physiology and Chronic Health Evaluation II (APACHE II) score ≥ 23 [8]. The choice of sedative could influence even the survival outcome in patients with sepsis.

Dexmedetomidine may improve survival through the attenuation of organ dysfunction in sepsis [5, 6]. However, the mechanisms underlying improved survival remain unclear and should be elucidated. This study aimed to determine which organ functions are improved using the sedation strategy with dexmedetomidine in the subgroup of patients with severe sepsis, in whom survival benefit was observed in the DESIRE trial.

## Methods

### Study design

This study is a post hoc subgroup analysis of the DESIRE trial [8], which was a multicenter randomized controlled trial conducted in eight ICUs in Japan. The DESIRE trial enrolled 201 patients with sepsis undergoing ventilation to assess the effects of a sedation strategy with dexmedetomidine (the DEX group) compared with that of a sedation strategy without dexmedetomidine (the non-DEX group). The protocol and results of the DESIRE trial have been reported elsewhere. The ethical review boards of all relevant institutions approved the study protocol, and all participants provided written informed consent prior to enrolment [8].

### Patients

In this subgroup analysis, we included the seriously ill patients among the 201 randomized patients in the DESIRE trial. Seriously ill patients were defined as those with APACHE II scores ≥ 23.

### Outcomes

As the primary outcome, we compared the trajectory of each organ component of the sequential organ failure assessment (SOFA) score between the groups. SOFA scores were obtained at 1, 2, 4, and 6 days after randomization. Next, we additionally analyzed the trajectories of the organs that were significantly different between groups. We evaluated the variables comprising each organ component of the SOFA score (e.g., urinary output and serum creatinine level as the renal component, total bilirubin level as the hepatic component, and the Glasgow coma score as the central nervous system component).

As secondary outcomes, we analyzed in-hospital mortality, 28-day mortality, renal replacement therapy, and ventilator-free days.

### Statistical analysis

Continuous variables are presented as the mean ± standard deviation or the median and interquartile range (IQR). Categorical variables are reported as frequencies and percentages (%). Continuous variables were compared using the *t* test or Wilcoxon rank-sum test and categorical variables using Fisher’s exact test between the DEX and non-DEX groups. A generalized linear model was used to examine the effect of dexmedetomidine on the natural logarithm of the daily urinary output and the serum creatinine concentration after excluding the chronic dialysis patients. All analyses were performed using JMP Pro version 13 (SAS Institute Inc., Cary, NC, USA) and SAS version 9.4 (SAS Institute Inc., Cary, NC). A *p* < 0.05 was considered statistically significant.

## Results

**Fig. 1:**
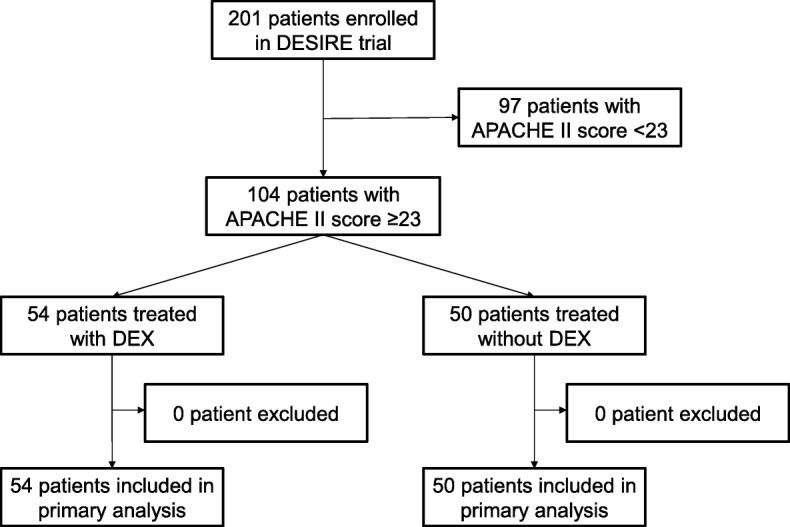
Flowchart of patients in this study. DESIRE, Dexmedetomidine for Sepsis in Intensive Care Unit Randomized Evaluation; APACHE, Acute Physiology and Chronic Health Evaluation; DEX, dexmedetomidine

Among the 201 patients enrolled in the DESIRE trial, we focused on the 104 patients with APACHE II scores ≥ 23 in this sub-study. Of these patients, 54 patients were sedated with dexmedetomidine and 50 patients without dexmedetomidine (Fig. 1).

Patients’ characteristics are shown in Table 1. There were no differences in APACHE II score between the groups, but the initial total SOFA scores in the DEX group were significantly lower than those in the non-DEX group (9 (7, 11) vs. 11 (9, 13); *p* = 0.01). The coagulation component of the SOFA score in the DEX group was significantly lower than that in the non-DEX group at the baseline. The daily dose of norepinephrine for patients with septic shock was not significantly different between the two groups (Additional file 1: Table S1).

**Table 1:** Patient characteristics

Data field	DEX group (*n* = 54)	non-DEX group (*n* = 50)	*P* value
Age, years, mean ± SD	70.7 ± 15.1	71.4 ± 13.2	0.80
Male subjects, n (%)	30 (56)	33 (66)	0.28
Body weight, kg, mean ± SD	53.5 ± 12.9	56.2 ± 13.8	0.30
APACHE II score, median (IQR)^a^	29 (25, 31)	30 (25, 33)	0.35
SOFA score, median (IQR)^b^	9 (7, 11)	11 (9, 13)	*0.01*
Respiratory SOFA score, median (IQR)	2 (1, 3)	2 (1, 3)	0.73
Cardiovascular SOFA score, median (IQR)	3 (2, 4)	3 (3, 4)	0.33
Neurological SOFA score, median (IQR)	1 (0, 3)	2 (0, 4)	0.47
Renal SOFA score, median (IQR)	2 (1, 2)	2 (0, 3)	0.64
Hepatic SOFA score, median (IQR)	0 (0, 1)	0 (0, 1)	0.36
Coagulation SOFA score, median (IQR)	0 (0, 2)	1 (0, 2)	*0.007*
Serum lactate level, mmol/L, median (IQR)^c^	3.9 (2.7, 6.4)	4.5 (3.0, 8.9)	0.19
Shock, n (%)^d^	33 (61)	33 (66)	0.69
Comorbidities			
Immunocompromised, n (%)	10 (19)	10 (20)	1.00
Hemodialysis, n (%)	3 (6)	5 (10)	0.48
Chronic respiratory disorder, n (%)	4 (7)	4 (8)	1.00
Chronic heart failure, n (%)	2 (4)	2 (4)	1.00
Site of infection			0.76
Abdomen, n (%)	21 (39)	17 (34)	
Thorax, n (%)	20 (37)	15 (30)	
Urinary tract, n (%)	3 (6)	7 (14)	
Skin and soft tissue, n (%)	4 (7)	4 (8)	
Other, n (%)	6 (11)	7 (14)	

*DEX* dexmedetomidine, *SD* standard deviation, *APACHE II* acute physiology and chronic health evaluation II, *SOFA* sequential organ failure assessment, *IQR* interquartile range

^a^The APACHE II score ranges from 0 to 71, with higher scores indicating severer disease.

^b^The SOFA score ranges from 0 to 24, with higher scores indicating more severe disease. The SOFA score consists of six organ-subscores ranges from 0 to 4.

^c^Serum lactate value was measured at randomization.

^d^Septic shock was defined as a SOFA score > 2 for the cardiovascular category and a lactate level > 2 mmol/L at randomization.

We showed *p*<0.05 in italic font.

The types of sedatives besides dexmedetomidine are shown in Additional file 2: Table S2. The number of patients with the administration of propofol in the DEX group was significantly lower than that in the non-DEX group on day 1. The number of patients with the administration of midazolam in the DEX group was significantly lower than that in the non-DEX group on days 1, 2, 3, 4, and 7.

The absolute changes in the SOFA score from the baseline are shown in Table 2. On day 4, the renal component of the SOFA score in the DEX group was significantly lower than that in the non-DEX group.

**Table 2:** The absolute change of sequential organ failure assessment score from the baseline

Data field	DEX group (*n* = 54)	Non-DEX group (*n* = 50)	*P* value
Day 2, *n* (%)	52 (96)	48 (96)	
Respiratory subscore, median (IQR)	0 (−1, 0)	0(−1, 1)	0.32
Cardiovascular subscore, median (IQR)	0 (0, 0)	0 (0, 0)	0.38
Neurological subscore, median (IQR)	0 (0, 1)	0 (0, 1)	0.87
Renal subscore, median (IQR)	0 (−1, 0)	0 (0, 0)	0.08
Hepatic subscore, median (IQR)	0 (0, 1)	0 (0, 0)	0.87
Coagulation subscore, median (IQR)	1 (0, 1)	0 (0, 1)	0.32
Total SOFA score, median (IQR)^a^	1 (−1, 2)	1 (0, 3)	0.20
Day 4, *n* (%)	43 (80)	41 (82)	
Respiratory subscore, median (IQR)	0 (−1, 0)	0 (−1, 1)	0.11
Cardiovascular subscore, median (IQR)	0 (−2, 0)	0 (−2, 0)	0.55
Neurological subscore, median (IQR)	0 (−1, 0)	0 (0, 0)	0.22
Renal subscore, median (IQR)	−1 (−1, 0)	0 (−1, 0)	**0.02**
Hepatic subscore, median (IQR)	0 (0, 1)	0 (0, 1)	0.97
Coagulation subscore, median (IQR)	1 (0, 2)	1 (0, 2)	0.89
Total score, median (IQR)	− 1 (−3, 2)	0 (−3, 3)	0.19
Day 6, *n* (%)	33 (61)	32 (64)	
Respiratory subscore, median (IQR)	0 (−2, 1)	0 (−1, 0)	0.93
Cardiovascular subscore, median (IQR)	− 1 (− 3, 0)	0 (−3, 0)	0.52
Neurological subscore, median (IQR)	0 (− 1, 0)	0 (0, 0)	0.37
Renal subscore, median (IQR)	− 1 (−1, 0)	− 1 (−1, 0)	0.23
Hepatic subscore, median (IQR)	0 (0, 1)	0 (0, 1)	0.91
Coagulation subscore, median (IQR)	1 (0, 2)	1 (0, 2)	0.78
Total score, median (IQR)	− 2 (−4, 2)	− 2 (−5, 1)	0.78

*DEX* dexmedetomidine, *SOFA* sequential organ failure assessment, *IQR* interquartile range

^a^The SOFA score ranges from 0 to 24, with higher scores indicating more severe disease. The SOFA score consists of six organ-subscores ranges from 0 to 4

**Fig. 2:**
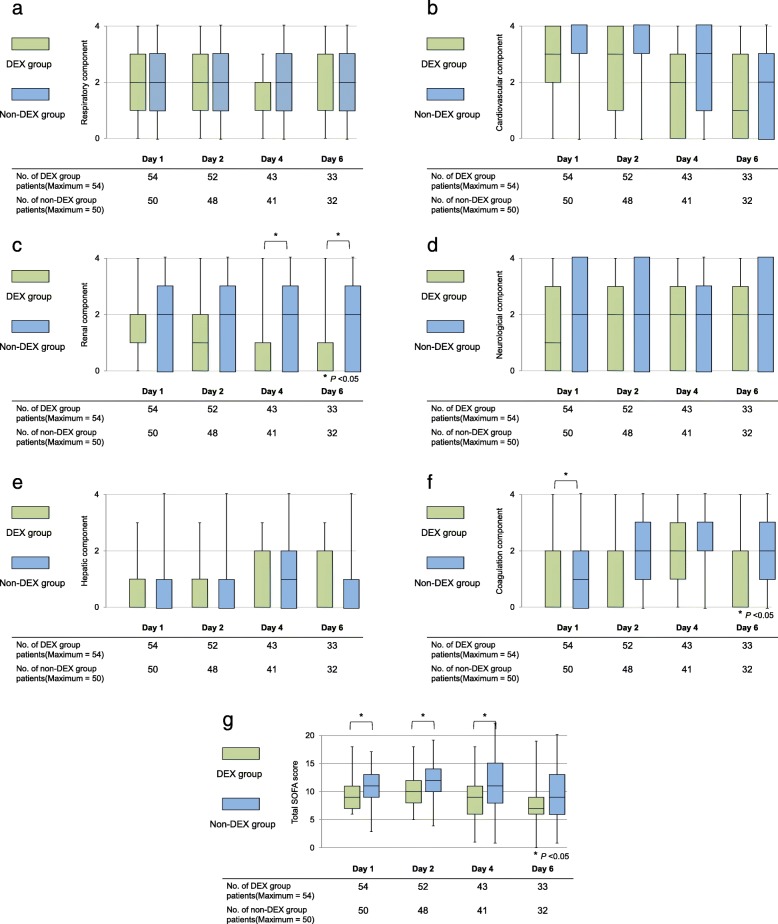
Comparison between the dexmedetomidine and non-dexmedetomidine groups regarding each component of the sequential organ failure assessment score. **a** Respiratory component. **b** Cardiovascular component. **c** Renal component. **d** Neurological component. **e** Hepatic component. **f** Coagulation component. **g** Total SOFA score. We compared continuous variables between both groups using Wilcoxon rank-sum test. DEX, dexmedetomidine

The renal components of the SOFA score in the DEX group were significantly lower than those in the non-DEX group on days 4 and 6 (-1(-1, 0) vs 0 (− 1, 0); *p* = 0.02) (Fig. 2).

**Fig. 3:**
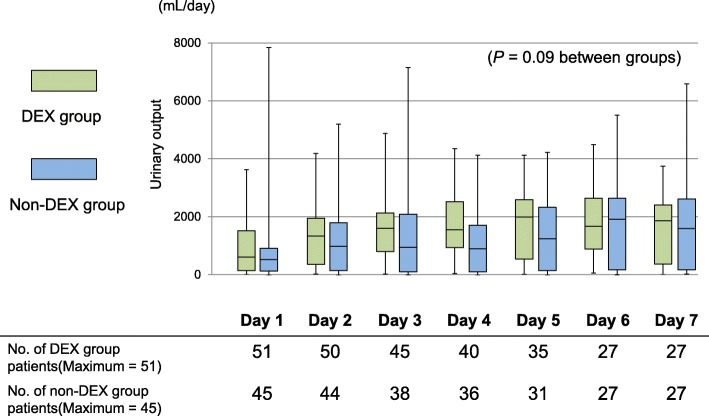
Comparison between the dexmedetomidine and non-dexmedetomidine groups regarding urinary output within a week. A generalized linear model was used to examine the effect of dexmedetomidine on the natural logarithm of the daily urinary output. DEX, dexmedetomidine

**Fig. 4:**
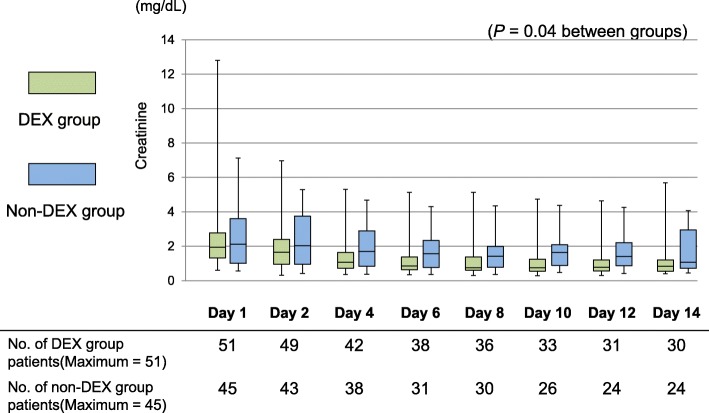
Comparison between the dexmedetomidine and non-dexmedetomidine groups regarding creatinine levels within a week. A generalized linear model was used to examine the effect of dexmedetomidine on the natural logarithm of the daily serum creatinine concentration. DEX, dexmedetomidine

Figures 3 and 4 show the daily urinary output and the serum creatinine concentrations in both groups. On day 1, no significant differences in daily urinary output and serum creatinine concentrations were observed between the groups. The daily urinary output during the first week did not differ significantly between the DEX group and the non-DEX group (*p* = 0.09). The serum creatinine concentrations during the first 2 weeks in the DEX group were significantly lower than those in the non-DEX group (*p* = 0.04) (Fig. 3). The number of patients with renal replacement therapy during the first week was not significantly different between the two groups (Additional file 3: Table S3).

The 28-day and in-hospital mortality rates in the DEX group were significantly lower than those in the non-DEX group (22% vs. 42%; *p* = 0.03, 28% vs. 52%; *p* = 0.01, respectively) (Table 3).

**Table 3:** Secondary outcomes

Data field	DEX group (*n* = 54)	Non-DEX group (*n* = 50)	*P* value
Renal replacement therapy in ICU, n (%)	27 (50)	29 (58)	0.41
Ventilator-free days, d, median (IQR)	18 (0, 23)	5 (0, 20)	0.09
28-day mortality, n (%)	12 (22)	21 (42)	*0.03*
Hospital mortality, n (%)	15 (28)	26 (52)	*0.01*

*DEX* dexmedetomidine, *ICU* intensive care unit, *IQR* interquartile range

We showed *p*<0.05 in italic font.

## Discussion

In this study, we found that the sedation strategy with dexmedetomidine was associated with an improvement in the renal component of the SOFA score in severely ill patients with sepsis. Additionally, the serum creatinine concentrations were lower in the DEX group than those in the non-DEX group.

The present study was the sub-analysis of the DESIRE trial, which did not show improvement in mortality statistically with the sedation strategy using dexmedetomidine in the entire cohort. However, it might have been underpowered for mortality and the sedation strategy with dexmedetomidine provided an 8% reduction in the 28-day mortality compared with no dexmedetomidine [8]. Furthermore, in the subgroup analysis that included severely ill patients (with APACHE II score ≥ 23), dexmedetomidine was associated with an improved survival rate (22% in the DEX group and 42% in the non-DEX group, *p* = 0.03). Therefore, we examined the mechanism underlying the improved mortality in severely ill patients treated with dexmedetomidine in this study.

Sepsis leads to organ dysfunction due to a systemic reaction to infection and results in a high mortality rate [9, 10]. Several studies have shown that septic acute kidney injury (AKI) is associated with a higher degree of illness severity and a higher mortality rate [11–13]. In the present study, dexmedetomidine was associated with an improved renal function in patients with sepsis and 28-day mortality in the DEX group was significantly lower than in the non-DEX group of patients with sepsis. However, we could not clarify the relationship between renal improvement with dexmedetomidine and survival benefit.

Recent reports have shown that renal inflammation, microcirculatory dysfunction, and apoptosis occur in sepsis [14–16]. Previous animal studies reported that dexmedetomidine prevented sepsis-induced AKI by regulating inflammation and apoptosis [17, 18]. Chung et al. reported that dexmedetomidine significantly reduced the levels of inflammatory cytokines, such as tumor necrosis factor-alpha and monocyte chemotactic protein-1, and ameliorated renal dysfunction among mice in the septic AKI model [17]. Additionally, Kai et al. reported that dexmedetomidine suppressed the expression of sepsis-induced inflammatory factors, such as tumor necrosis factor-alpha and interleukin-6, and reduced tubular apoptosis in mice [18]. Dexmedetomidine was also reported to reduce the level of norepinephrine in the blood, resulting in an increase in renal blood flow and urinary output [19]. The results of the present study suggest that dexmedetomidine might improve sepsis-induced AKI through the attenuation of an excessive inflammatory response or sympathetic tone. However, we could not confirm this hypothesis because we could not evaluate the inflammatory response in the present study.

Previous randomized controlled trials reported that dexmedetomidine might attenuate renal injury during the perioperative period in patients who underwent cardiac surgery [20–22]. Zhai et al. showed that dexmedetomidine reduced the levels of serum urea nitrogen, creatinine, and neutrophil gelatinase-associated lipocalin after cardiac valve replacement surgery under cardiopulmonary bypass [20]. However, no randomized controlled trials have reported that dexmedetomidine improves renal function among patients with sepsis. Future studies are needed to confirm our results regarding the improvement of renal function in patients with sepsis.

Our study has several limitations. The most critical limitation is that this study is a post hoc subgroup analysis enrolling with higher APACHE II score that results in the differences in baseline characteristics between groups. Conceptually, the randomized design of this study could also balance baseline characteristics in this subgroup. Therefore, these differences between groups might occur by chance. In addition, because the sample size was not adequately large to conduct multivariable analyses, we did not adjust the baseline characteristics. In fact, the initial total SOFA score and coagulation SOFA score were significantly higher in the non-DEX group than those in the DEX group. More severe coagulation abnormality might increase the mortality in the non-DEX group which was reported in a previous study [23]. However, at least, the difference of the initial coagulation SOFA score did not directly influence the evaluation of the renal component of the SOFA score, because the initial renal component of the SOFA score did not differ. Therefore, the results of our study should be interpreted as hypothesis-generating which should be confirmed through future studies. Second, our study was a post hoc analysis and applied multiple comparisons for exploratory purposes. The difference in the renal component might be due to chance. It is necessary to evaluate our findings in further well-designed studies. Third, we could not evaluate the mechanisms in other organs besides those included in the SOFA score. The SOFA score was developed to provide a rough assessment of each organ dysfunction; therefore, we could not detect minor organ dysfunctions using the SOFA score. We may, therefore, have missed identifying the mechanism underlying the improved outcome, besides renal protection. For instance, dexmedetomidine was reported to modulate inflammation or liver dysfunction [4–6]. Fourth, we collected and analyzed the data of absolute changes in each organ component of the SOFA score only in 60% of the patients on day 6 in both groups as shown in Table 2. Because the data of deceased patients were excluded and data of patients discharged from ICU were missing, the number of patients’ data on day 6 decreased. Therefore, it might not be appropriate to conclude that dexmedetomidine affected organ dysfunction in the DEX group.

## Conclusions

The sedative strategy with dexmedetomidine for severely ill patients with sepsis improves renal dysfunction and mortality rate. To reiterate, our study is an exploratory and hypothesis-generating study and our findings need to be confirmed in future studies.

## Supplementary information

**Additional file 1: Table S1.** The daily dose of norepinephrine among 66 patients septic shock between two groups during the first week.

**Additional file 2: Table S2.** The daily usage of propofol and midazolam between two groups during the first week.

**Additional file 3: Table S3.** The number of patient received renal replacement therapy between two groups during the first week

## Data Availability

The datasets generated and analyzed during the current study are not publicly available because of privacy concerns and institutional policy.

## Abbreviations

AKI: Acute kidney injury
APACHE: Acute Physiology and Chronic Health Evaluation
DESIRE: Dexmedetomidine for Sepsis in Intensive Care Unit Randomized Evolution
DEX: Dexmedetomidine
ICU: Intensive care unit
SOFA: Sequential organ failure assessment
