# Supplementary material for: Dexmedetomidine improved renal function in patients with severe sepsis: an exploratory analysis of a randomized controlled trial
Source: J Intensive Care. 2020 Jan 2;8:1. doi: 10.1186/s40560-019-0415-z (PMC6939335; doi:10.1186/s40560-019-0415-z)
Supplement: Supplementary file 1 — Additional file 1: Table S1. The daily dose of norepinephrine among 66 patients septic shock between two groups during the first week. [file 40560_2019_415_MOESM1_ESM.doc]

Table S1. The daily dose of norepinephrine among 66 patients septic shock between two groups during the first week

| Data field | DEX group  (n=33) | non-DEX group (n=33) | *P* value |
| --- | --- | --- | --- |
| Day 1, n=66^a^ | n=33 | n=33 |  |
| Dose, median (IQR), mg | 5.4 (2.5, 8.6) | 6.8 (4.3, 13.4) | 0.10 |
| Day 2, n=66 | n=33 | n=33 |  |
| Dose, median (IQR), mg | 7.3 (3.8, 16.9) | 9.0 (3.1, 22.3) | 0.37 |
| Day 3, n=58 (missing data n=8)  Dose, median (IQR), mg | n=30  5.1 (0, 12.8) | n=28  7.4 (2.0, 19.1) | 0.21 |
| Day 4, n=56 (missing data n=10)  Dose, median (IQR), mg | n=28  0.9 (0, 7.8) | n=28  3.4 (0, 9.0) | 0.79 |
| Day 5, n=54 (missing data n=12) | n=27 | n=25 |  |
| Dose, median (IQR), mg | 0 (0, 3.8) | 0 (0, 3.5) | 0.85 |
| Day 6, n=50 (missing data n=16) | n=26 | n=24 |  |
| Dose, median (IQR), mg | 0 (0,1.1) | 0 (0, 0.8) | 0.86 |
| Day 7, n=50 (missing data n=16) | n=26 | n=24 |  |
| Dose, median (IQR), mg | 0 (0, 0) | 0 (0, 0.2) | 0.42 |

DEX: dexmedetomidine, IQR: interquartile range

a Septic shock was defined as a cardiovascular component of the sequential organ failure assessment score >2 and a serum lactate concentration >2 mmol/l at randomization. 38 patients without shock patients were excluded from this analysis.
