# Supplementary material for: Dexmedetomidine improved renal function in patients with severe sepsis: an exploratory analysis of a randomized controlled trial
Source: J Intensive Care. 2020 Jan 2;8:1. doi: 10.1186/s40560-019-0415-z (PMC6939335; doi:10.1186/s40560-019-0415-z)
Supplement: Supplementary file 2 — Additional file 2: Table S2. The daily usage of propofol and midazolam between two groups during the first week. [file 40560_2019_415_MOESM2_ESM.doc]

Table S2. The daily usage of propofol and midazolam between two groups during the first week

| Data field | DEX group | non-DEX group | *P* value |
| --- | --- | --- | --- |
| Day 1, n=104 | n=54 | n=50 |  |
| Propofol, n, (%)  Midazolam, n, (%) | 12 (22)  10 (19) | 21 (42)  20 (40) | **0.036**  **0.018** |
| Day 2, n=102 (missing data n=1) | n=53 | n=49 |  |
| Propofol, n, (%)  Midazolam, n, (%) | 12 (23)  6 (11) | 20 (41)  21 (43) | 0.057  **0.0006** |
| Day 3, n=89 (missing data n=5)  Propofol, n, (%)  Midazolam, n, (%) | n=47  13 (28)  3 (6) | n=42  14 (33)  16 (38) | 0.65  **0.0005** |
| Day 4, n=81 (missing data n=9)  Propofol, n, (%)  Midazolam, n, (%) | n=41  10 (24)  1 (2) | n=40  12 (30)  13 (33) | 0.62  **0.0003** |
| Day 5, n=71 (missing data n=15) | n=36 | n=35 |  |
| Propofol, n, (%)  Midazolam, n, (%) | 8 (22)  3 (8) | 10 (29)  9 (26) | 0.59  0.063 |
| Day 6, n=59 (missing data n=25) | n=28 | n=31 |  |
| Propofol, n, (%)  Midazolam, n, (%) | 6 (21)  4 (14) | 11 (35)  8 (26) | 0.26  0.34 |
| Day 7, n=59 (missing data n=24) | n=28 | n=31 |  |
| Propofol, n, (%)  Midazolam, n, (%) | 5 (18)  2 (7) | 5 (16)  10 (32) | 1.00  **0.023** |

DEX: dexmedetomidine,
