# Supplementary material for: Dexmedetomidine improved renal function in patients with severe sepsis: an exploratory analysis of a randomized controlled trial
Source: J Intensive Care. 2020 Jan 2;8:1. doi: 10.1186/s40560-019-0415-z (PMC6939335; doi:10.1186/s40560-019-0415-z)
Supplement: Supplementary file 3 — Additional file 3: Table S3. The number of patient received renal replacement therapy between two groups during the first week [file 40560_2019_415_MOESM3_ESM.doc]

Table S3. The number of patient received renal replacement therapy between two groups during the first week

| Data field | DEX group | non-DEX group | *P* value |
| --- | --- | --- | --- |
| Day 1, n=104 | n=54 | n=50 |  |
| Number of patients received RRT, n (%) | 16 (30) | 16 (32) | 0.83 |
| Day 2, n=102 (missing data n=1) | n=53 | n=49 |  |
| Number of patients received RRT, n (%) | 21 (40) | 25 (51) | 0.32 |
| Day 3, n=89 (missing data n=5)  Number of patients received RRT, n (%) | n=47  17 (36) | n=42  20 (48) | 0.29 |
| Day 4, n=81 (missing data n=9)  Number of patients received RRT, n (%) | n=41  14 (34) | n=40  18 (45) | 0.37 |
| Day 5, n=71 (missing data n=15) | n=36 | n=35 |  |
| Number of patients received RRT, n (%) | 11 (31) | 18 (51) | 0.09 |
| Day 6, n=59 (missing data n=25) | n=28 | n=31 |  |
| Number of patients received RRT, n (%) | 11 (39) | 14 (45) | 0.79 |
| Day 7, n=59 (missing data n=24) | n=28 | n=31 |  |
| Number of patients received RRT, n (%) | 9 (32) | 13 (42) | 0.59 |

DEX: dexmedetomidine, RRT: renal replacement therapy
